# 2640. Estimated Additional Burden Averted from Use of Cell-Based Influenza Vaccines Compared to Egg-Based Influenza Vaccines Among People 0-64 Years of Age in the United States: 2017-2020 Seasons

**DOI:** 10.1093/ofid/ofad500.2252

**Published:** 2023-11-27

**Authors:** Ian McGovern, Alexandra Taylor, Aditya Sardesai, Hector Toro-Diaz, Mendel Haag

**Affiliations:** CSL Seqirus, Waltham, Massachusetts; Evidera, San Francisco, California; Evidera, San Francisco, California; Evidera, San Francisco, California; CSL Seqirus, Waltham, Massachusetts

## Abstract

**Background:**

Influenza vaccines have traditionally been produced in eggs, which can introduce egg-adaptive mutations. Cell-based influenza vaccines avoid egg-adaptive mutations, potentially improving match to circulating influenza viruses and therefore vaccine effectiveness. This study modeled the public health impact if all vaccinated people 0-64 years of age in the United States received cell-propagated inactivated quadrivalent influenza vaccine (IIV4c) compared to egg-propagated inactivated quadrivalent influenza vaccine (IIV4) across the 2017-2018 through 2019-2020 Influenza Seasons.

**Methods:**

The modeling method used by the US Centers for Disease Control and Prevention (CDC) for estimating overall burden averted due to influenza vaccination was extended to a relative vaccine effectiveness (rVE) context. The model utilized CDC data on influenza vaccine uptake, influenza incidence, influenza-related healthcare resource use and deaths. CDC estimates of absolute vaccine effectiveness (aVE) (any vaccine) were used as the aVE of IIV4. Based on previously published rVE estimates generated from the same real-world database, the rVE of IIV4c vs IIV4 was assumed to be 19.3% (95% Confidence interval: 9.5 to 28) in 2017-2018, 7.6% (6.5 to 8.6) in 2018-2019, and 17.2% (15.8 to 18.6) in 2019-2020. Base-case results were validated with deterministic (DSA) and probabilistic (PSA) sensitivity analyses.

**Results:**

**Figure 1** shows the anticipated number influenza-related cases and complications that would be averted if all vaccinated people 0-64 years of age received IIV4c or IIV4. Across the 3 influenza seasons, use of IIV4c would result in prevention of an additional 12,706,837 symptomatic illnesses, 6,222,820 outpatient visits, 80,390 hospitalizations, and 2,765 deaths. DSA results showed that the rVE estimate was associated with the most variability while the burden estimates were most influential in the other two seasons. Mean PSA results were all within a 0.5% difference of base-case results.

**Figure 1.**
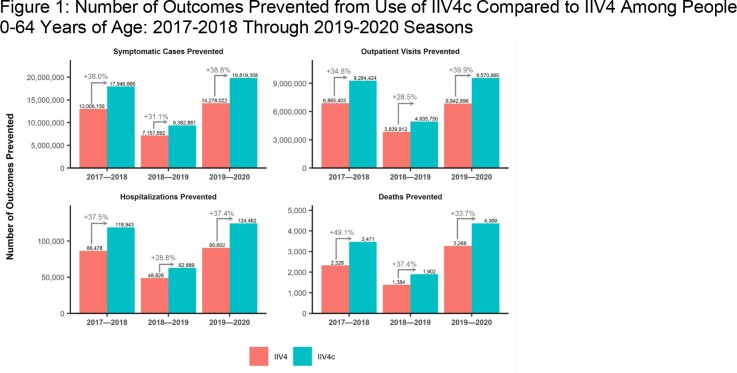
Number of Outcomes Prevented from Use of IIV4c Compared to IIV4 Among People 0-64 Years of Age: 2017-2018 Through 2019-2020 Seasons

**Conclusion:**

Use of IIV4c instead of IIV4 during the 2017-2020 influenza seasons in the US would have had a substantial public health impact due to a 28.5% to 49.1% increase in influenza cases prevented.

**Disclosures:**

**Ian McGovern, MPH**, CSL Seqirus: Employment|CSL Seqirus: Stocks/Bonds **Alexandra Taylor, MSc**, CSL Seqirus: Advisor/Consultant **Aditya Sardesai, MSc**, CSL Seqirus: Advisor/Consultant|Merck & Co. Inc: Grant/Research Support|Merck & Co. Inc: Grant/Research Support **Hector Toro-Diaz, PhD**, CSL Seqirus: Advisor/Consultant **Mendel Haag, PhD, PharmD**, CSL Seqirus: Stocks/Bonds

